# A Review of the Therapeutic Potential of Stem Cell‐Derived Nanocarriers to Treat Neurological Disorders

**DOI:** 10.1002/open.202500425

**Published:** 2026-08-20

**Authors:** Nnamdi Ikemefuna Okafor, Ahmed Abdelgader, Magdi Abobaker, Yahya E. Choonara

**Affiliations:** ^1^ Wits Advanced Drug Delivery Platform Research Unit Department of Pharmacy and Pharmacology School of Therapeutic Sciences Faculty of Health Sciences University of the Witwatersrand Johannesburg South Africa

**Keywords:** blood brain barrier, exosomes, extracellular vesicles, nanocarriers, neurological diseases, stem cells

## Abstract

Neurological disorders (NDs) are characterized by substantial loss of specific neurons, with Alzheimer's and Parkinson's diseases being the most frequent NDs and nearly 99% of all “foreign substances” are prohibited from entering the brain by the blood‐brain barrier (BBB) and the blood‐cerebrospinal fluid barrier (CFB). These barriers, while crucial for brain protection, pose significant challenges for drug delivery, as they restrict the entry of many therapeutic agents into the brain, and this represents the primary manifestation of the absence of pathogenesis‐targeting therapeutics. With significant success across multiple cell transplantation research efforts, stem cell therapy has been utilized for decades to treat neurological disorders. They work by replacing injured or lost cells directly, releasing proliferation and neurotrophic factors through autocrine and paracrine actions, suppressing neurological inflammation, and activation of endogenous brain progenitor cells. Nanocarriers derived from stem cells represent an innovative and promising therapeutic strategy for combating neurological diseases, combining faculties of regeneration of stem cells with the precision of nanotechnology. These nanocarriers possess natural biocompatibility and can efficiently cross the BBB to transport the therapeutic agents directly to affected neural tissues, thereby promoting enhanced treatment efficacy while reducing off‐target effects. However, key challenges remain in large‐scale production, standardization, and long‐term safety. Thus, this review has examined the potential applications of stem cell extracellular vesicle (EV)‐nanocarriers, mainly exosomes and recent development in the treatment of neurological diseases.

## 1 Introduction to Common Neurological Disorders

Neurological diseases (NDs) encompass a wide range of conditions affecting the brain, spinal cord, and nerves, leading to diverse debilitating symptoms [1]. NDs, such as Alzheimer's disease (AD), Parkinson's disease (PD), and Huntington's disease (HD), are becoming a rising and challenging health concern worldwide, particularly among the ageing population in the world [2]. These conditions are characterized by a gradual loss of neurons, resulting in motor, sensory, and cognitive impairments [3]. They share the pathogenic buildup of misfolded proteins, including amyloid‐β in AD and α‐synuclein in PD, activating a series of pathogenic pathways that include oxidative stress, mitochondrial malfunction, and inflammation, which lead to neuronal cell death [4].

AD is most prevalent, accounting for 60%–70% of all dementia cases globally and affecting around 50 million individuals [5]. AD is a significant public health and financial burden, with annual expenses surpassing one trillion US dollars and anticipated to climb by 2030 [6, 7]. This disease is characterized by a progressive drop in cognitive abilities, memory impairment, and motor disability, which is mainly triggered by selective loss of neurons in vital regions of the brain, such as the neocortex, hippocampus, amygdala, locus coeruleus, and nucleus basalis of Meynert [8, 9]. AD is distinguished by the formation of Aβ plaques and neurofibrillary tangles composed of hyperphosphorylated tau protein. These aberrant aggregates disrupt neuronal function and synaptic integrity, causing neurodegeneration [4, 10, 11]. These pathologic processes are thought to be initiated years before the clinical symptoms emerge, with the Aβ‐Tau‐Neurodegeneration (A/T/N) biomarker framework currently employed to identify AD at its preclinical stage. Despite extensive attempts to elucidate AD pathophysiology, therapies are limited, with only five FDA‐approved drugs, all of which provide symptomatic but not disease‐modifying or disease‐halting benefits [6].

PD is a progressive disorder affecting approximately 1% of individuals over the age of 65 globally [11, 12]. James Parkinson first described the disease back in 1817 in his work titled “An Essay on the Shaking Palsy” [3]. PD is characterized by the specific degeneration of dopamine‐producing neurons in the substantia nigra pars compacta, with a substantial decrease in striatal dopamine levels, generally in excess of 80% [9]. The neuronal loss is associated with the aggregation of α‐synuclein protein, which leads to the formation of Lewy bodies and Lewy neurites in nigrostriatal neurons and other affected parts of the central nervous system (CNS), thereby interfering with brain function and facilitating disease progression [4, 8]. Though the exact causes of PD are not yet clear, research shows that oxidative stress and cytotoxicity are key mechanisms in inducing damage in the dopaminergic neurons [8]. Clinically, patients with PD demonstrate a variety of motor symptoms, such as resting tremors, bradykinesia, muscle rigidity, and postural instability. Aside from these, nonmotor symptoms like cognitive impairment, sleep difficulties, depression, and anxiety have a significant impact on quality of life [9, 10, 13, 14]. Although the understanding of PD pathophysiology has advanced, there remains no treatment that can halt or reverse the ongoing loss of dopamine‐producing neurons. This is crucial for research to continue into targeted therapies that can slow neurodegeneration and improve long‐term outcomes for patients with PD [11].

HD is a neurodegenerative condition defined by motor impairment, psychological instabilities, and cognitive deterioration, and it follows an autosomal‐dominant inheritance pattern. HD is relatively rare, with an approximate occurrence of 1 in 10,000 individuals of Western European descent [11]. Symptoms typically arise during adulthood, around the fourth or fifth decade of life; however, individuals as young as 10 years may, in exceptional circumstances, experience the onset of the disease [14]. HD develops from an aberrant expansion of CAG trinucleotide repeats within the huntingtin (HTT) gene, leading to the emergence of a faulty HTT protein (mHTT) with excessively long polyglutamine stretches [4]. While this protein is present throughout the body, neurodegeneration in HD is highly selective, with the striatum being the most severely affected region. As the disease progresses, other areas, including the cerebral cortex, also experience neuron cell loss [4, 9]. The clinical features of HD can be underscored by the progressive neural degeneration. These include weight loss, sleep disturbances, and personality changes. As the disease progresses, individuals develop chorea, a movement disorder characterized by involuntary, irregular movements along with neuropsychiatric symptoms that further impact daily function. These combined effects lead to severe disability and, ultimately, increased mortality [8, 9, 15].

A stroke is a potentially fatal medical event that occurs when blood supply to the brain is unexpectedly interrupted, either by a blocked artery (ischemic stroke) or by an artery rupture (hemorrhagic stroke) [13]. Among these, ischemic stroke is the most frequent, accounting for most instances. It is the third largest cause of death in the United States and the leading cause of disability in adults, culminating in high societal and economic costs. Each year, approximately 600,000 individuals experience an ischemic stroke, with 30% not surviving and another 30% suffering from permanent disability [11]. Ischemic strokes can be further classified into global ischemia and focal ischemia, depending on how blood flow is disrupted. Global ischemia happens following transient cardiac arrest and is histologically characterized by delayed neuronal death, while glial cells are often spared. Global ischemia can be lethal in humans after as little as 10 min at normal body temperature. Focal ischemia, on the other hand, results from a temporary or permanent restriction in blood flow within the territory of a cerebral artery, which can be frequently triggered by embolic or thrombotic blockage. This form of ischemia results in pannecrosis, which encompasses the death of all cellular constituents, involving neurons, glial, and vascular cells of the brain [8]. Despite extensive research, most of the advanced clinical trials for treating ischemic stroke interventions have not resulted in efficacious therapeutic strategies. Despite enhanced knowledge of the pathogenesis of these disorders, therapeutic intervention remains restricted, and therefore, the urgency to continue investigating new effective treatments is even more compelling [5]. This serves to emphasize the critical necessity of new research based on preserving brain cells, saving neurons, and restoring damaged tissue to avert the damaging effect of stroke [11].

## 2 Challenges in the Treatment of Neurological Disorders Arising From BBB Complexity

**FIGURE 1:**
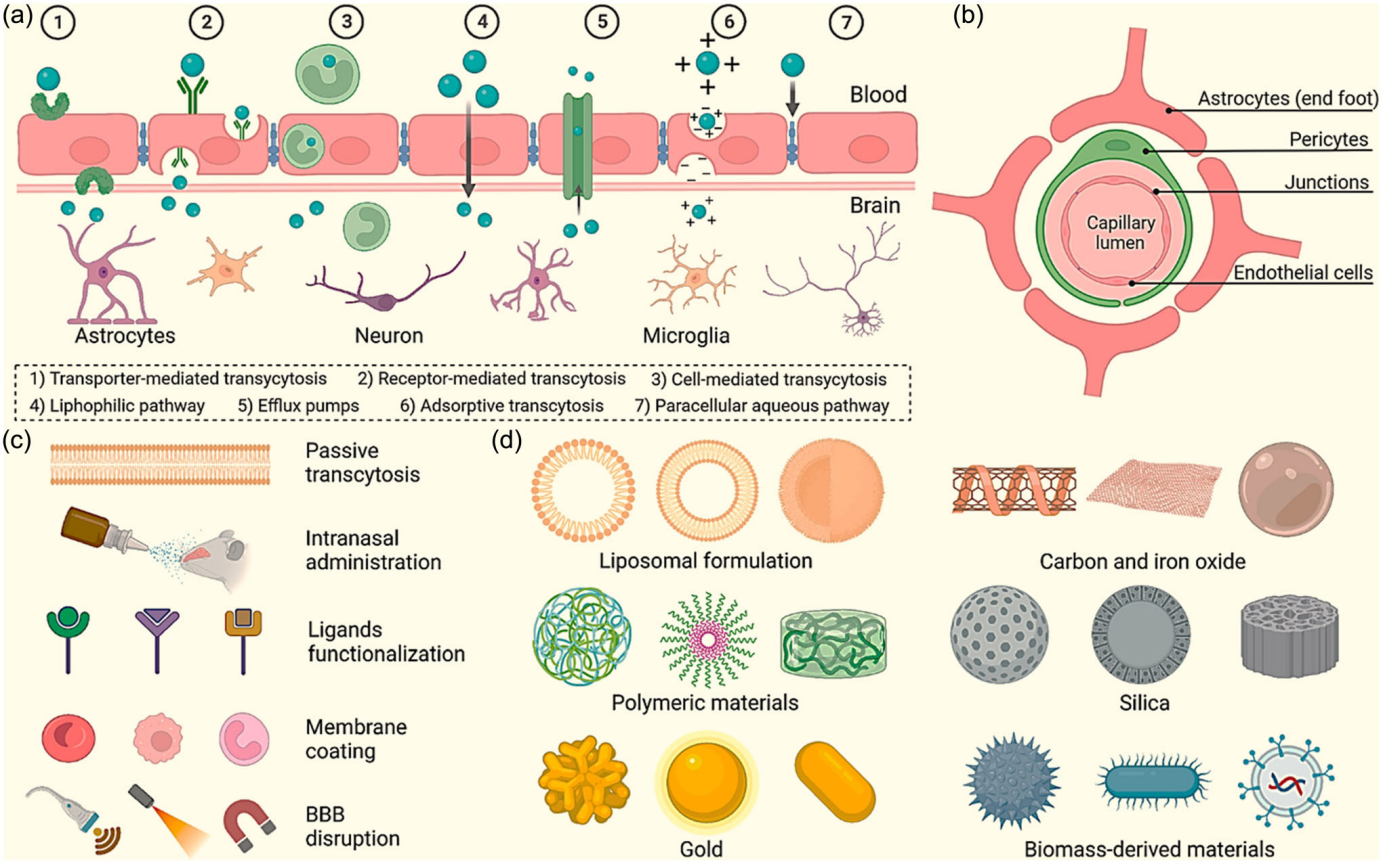
Illustration of (a) different mechanisms of BBB penetration; (b) structure of BBB; (c) noninvasive techniques to enhance BBB permeability and drug delivery; and (d) engineered nanocarriers designed for precise brain targeting. Adapted from [17].

The blood brain barrier (BBB) is an extremely selective semipermeable structure made up of brain endothelial cells, pericytes, astrocytes, and the basement membrane, all of which are components of the neurovascular system [16]. This system rigorously regulates the admission of oxygen and nutrients into the brain while allowing for waste disposal and limiting the entry of dangerous chemical substances (Figure 1) [18, 19, 20]. Though the BBB serves a critical function in maintaining a consistent microenvironment in the brain, its intricacy and selective permeability impede the transport of therapeutic medications into the brain, limiting therapy choices for brain disorders [21, 22, 23]. The BBB's tight connections are efficient barriers that inhibit about 98% of small molecules and nearly all large molecules from entering the brain, making drug delivery challenging [24]. For example, in AD, many drugs fail to cross the BBB during clinical trials, while in PD, the delivery of neuroprotective drugs to deeper brain regions like the substantia nigra is a major obstacle [25, 26]. Additionally, BBB integrity can also be modified during disease states to enhance or decrease permeability, further making drug delivery more complex [27, 28]. To mitigate this, more effective drug delivery systems, such as nanoparticles, liposomes, dendrimers, micelles, and carbon nanotubes, have been investigated since they have a great deal of potential to accelerate drug transport and enable targeted delivery to specific brain regions [24, 29]. Physical techniques, like focused ultrasound and microbubble‐assisted delivery, can temporarily interrupt the BBB to allow drug passage, while chemical strategies employ permeation enhancers or receptor‐mediated transport to enhance nanocarrier uptake [30, 31]. These innovative approaches have the potential to transform conventional drug delivery methods, promote treatment effectiveness, and minimize side effects, ultimately benefiting patients with neurological disorders.

## 3 Role of Nanotechnology in Drug Delivery

**FIGURE 2:**
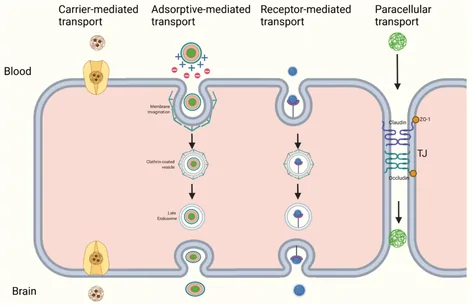
Schematic depiction of nanocarrier transport mechanisms across the BBB. Adapted from [32].

**FIGURE 3:**
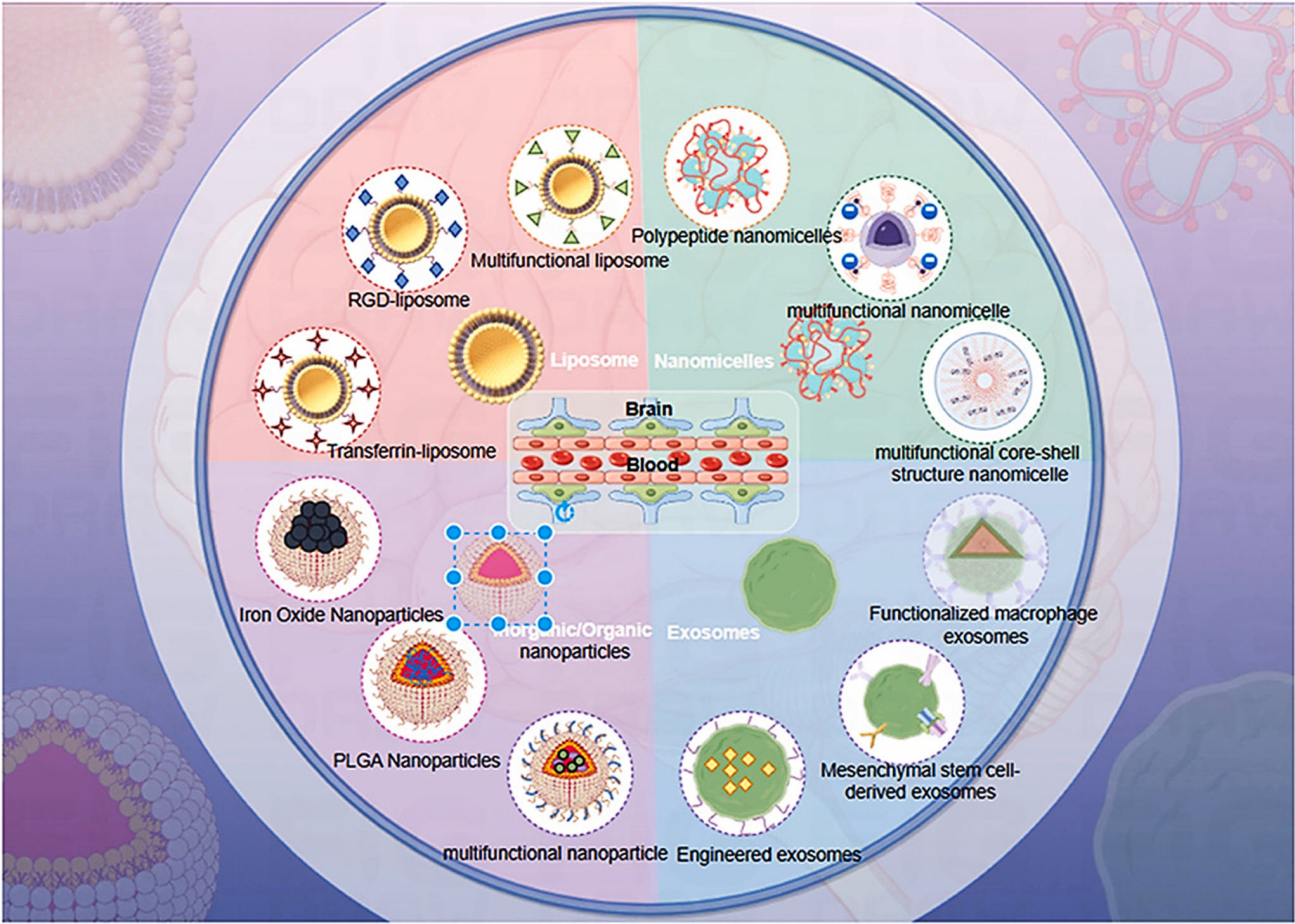
Nanotechnology‐enabled approaches for overcoming the BBB in central nervous system disorders. Adapted from [33].

Nanotechnology is a multidisciplinary science that combines chemistry, physics, engineering, and biology to manipulate matter on the nanoscale (1–100 nm) and produce materials with defined physicochemical features. It has crucial applications in drug delivery, in particular in overcoming the challenges posed by the BBB in the management of neurological disorders. By controlling the size, surface charge, and functionalization of nanoparticles, it makes it possible to deliver drugs across the BBB (Figure 2) [24, 30]. For example, polymeric nanoparticles, liposomes, and dendrimers can be designed to carry drugs, proteins, or nucleic acids, enabling controlled release and better therapeutic efficacy in the CNS (Figure 3) [24]. Furthermore, surface ligand modification of nanoparticles with antibodies or peptides specifically targeted against biomarkers found on diseased neural cells, thereby promoting site specificity and minimized side effects [34]. Moreover, nanomaterials can be utilized for imaging; hence, the real‐time observation of their migration can be achieved via techniques such as magnetic resonance and fluorescence imaging [2, 35]. However, there remain challenges to be addressed, such as ensuring targeting accuracy, biocompatibility, minimizing toxicity, and production on a large scale [22, 36]. Overall, nanotechnology represents a revolutionary approach to CNS drug delivery that permits the development of precise, noninvasive protocols that enhance therapeutic efficacy, permit advanced monitoring, and ultimately accelerate treatment outcomes in patients with neurological disorders.

## 4 Stem Cell‐Base Treatments

Stem cells represent a distinct class of cells that exhibit two important abilities: differentiation and self‐renewal aptitude. These cells can be extracted from tissues and can be guided to differentiate into specific cell types by controlling the physicochemical microenvironments [37]. Although they hold promise to treat disease, most of the clinical evidence supporting stem cell therapies originates from small, randomized clinical trials with often limited therapeutic benefit. Moreover, stem cell‐based treatments are also limited by factors including immune rejection and the potential risk of malignant transformation. Accordingly, significant hurdles remain, and the anticipated therapeutic efficacy has yet to be fully realized [38]. Recent progress in stem cell science has led to innovative ways of delivering stem cells, mitigating their limitations while maintaining the therapeutic benefits. One approach uses stem cell membrane‐coated nanoparticles, that retain the natural surface features of stem cells to target specific areas. Another exciting development is using stem cell‐derived extracellular vesicles (EVs), which can carry therapeutic material without the risks of cancer or immune rejection. Additionally, developing delivery methods that use stem cells embedded in scaffolds and scaffold‐free stem cell sheets have shown promise for facilitating tissues to grow and regenerate [39]. Stem cell membranes and EVs have come to be a central player in stem cell therapy and are an intrinsic type of nanoparticle capable of delivering biomolecules such as proteins, nucleic acids, and lipids to other cells. Their inherent biocompatibility, coupled with targetability, makes it desirable over artificial nanocarriers, which may have limitations like poor degradation within the body, immune reaction against them, and toxicity. These cell‐derived nanocarriers have several benefits in drug delivery, including longer in vivo circulation times, the ability to penetrate the BBB, high stability, and low risk of triggering immune responses, and are therefore desirable for targeted drug delivery. However, despite the high promise of stem cell‐derived nanocarriers, their application in clinical settings is a major challenge. These include low extraction yields, purity, batch variability, and low efficiency in drug loading, hindering large‐scale production and standardization of use. To address these shortfalls, bionic EV‐mimetic nanovesicles have been developed to replicate natural EVs’ key features while offering easier production [38].

### 4.1 Stem Cells in Nanotechnology

Stem cells have enormous opportunities in regenerative medicine since they can initiate repair and replace damaged tissues. This has the potential to revolutionize disease treatment interventions. However, there are also significant challenges linked to stem cell treatments, involving immune rejection, tumor risk, and the need for precise control of stem cell differentiation. These issues need to be addressed through novel methods to ensure safety, efficacy, and scalability [40]. Stem cells can be more effective when combined with nanotechnology and have resulted in the emergence of stem cell nanotechnology, which combines the regenerative potential of stem cells with the precision of nanotechnology. This field uses stem cells, along with their membranes, EVs, and secretomes, to promote drug delivery systems, ensuring targeted and efficient treatment [41]. For example, stem cell‐derived EVs can be designed to deliver therapeutic substances like drugs, proteins, or genetic material to specific cells or tissues, thereby improving the precision of treatments and minimizing unwanted effects.

### 4.2 EVs Classification

**FIGURE 4:**
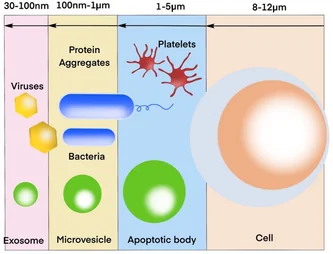
Comparative visualization of EV subtypes, incorporating exosomes, microvesicle, apoptotic bodies relative to viruses, bacteria, and platelets, with size progression shown from nanoscale vesicles to cellular dimensions. The figure illustrates hierarchical arrangement of these biological components, which shows the size scale from exosomes to cells. Adapted from [44].

EVs are minute heterogeneous membrane structures that play a crucial role in communication between cells. They are found in a variety of bodily fluids, including plasma, urine, cerebral fluid, synovial fluid, amniotic fluid, saliva, milk, and even the media of in vitro grown cell lines. EVs were first recognized by Wolf as “platelet dust” and initially regarded as cellular debris with no significant function [7, 42]. However, a breakthrough study in 1996 revealed that EVs could influence immune responses, basically altering the perception of their biological relevance [43]. EVs are divided into three groups (Table 1) depending on their size and formation: exosomes (40–150 nm), microvesicles (MVs; 100–1000 nm), and apoptotic bodies (ABs; 800–5000 nm) (Figure 4) [7, 42]. Nevertheless, this overlap in size and density, makes it technically challenging to isolate and characterize each type individually [43]. EVs are enclosed by a lipid bilayer membrane that contains several proteins and envelopes a range of compounds, including RNA and soluble proteins. These molecular payloads mediate cell communication. The surface of EVs is coated with particular receptors, which allow them to be detected by acceptor cells and transfer proteins, lipids, and genetic material, thereby regulating the physiological state of the acceptor cells. Notably, the molecular payload of EVs reflects the state and health of the parent cells, rendering them useful biomarkers for disease and potential agents for therapeutic interventions [7, 49]. EVs are currently being used directly or designed for therapy, such as regenerative medicine, cancer, and immune system modulation [50].

**TABLE 1:** Classification of EVs based on size and formation with their biomarkers, potential, and limitations.

EVs type	Typical size range	Biogenesis/formation mechanism	Key cargo (molecular components)	Surface markers	Advantages	Limitations	References
Exosomes	30–150 nm (up to 200 nm)	Inward budding of endosomal membrane to form multivesicular bodies	miRNAs, mRNAs, proteins (cytosolic/endosomal), lipids (ceramide)	CD63, CD81, CD9, Alix, TSG101, Flotillin	High stability in circulation; ability to cross the BBB; low immunogenicity.	Difficult to isolate with high purity; low yield from cell culture; overlapping size with smaller MVs (heterogeneity).	[37, 42, 44, 45]
MVs	100–1000 nm (up to 2 µm)	Outward budding and scission of the plasma membrane	miRNAs, mRNAs, proteins (cytosolic/endosomal), lipids (ceramide)	Annexin V, integrins, selectins, phosphatidylserine	Actively carry bioactive enzymes; high capacity for rapid intercellular communication and signaling	High heterogeneity in size and cargo; less defined molecular markers compared to exosomes; rapid clearance	[46, 47, 48]
ABs	1–5 µm (500 nm–5 µm)	Plasma membrane blebbing during programed cell death	Nuclear fragments (DNA/histones), organelles, cytoplasmic components	Annexin V, phosphatidylserine, histone	Act as markers of cell death and assist in safe clearance of cellular debris by phagocytes.	Rapidly cleared by phagocytes; high variability; difficult to isolate and standardize; usually only produced in dying cells.	[37, 38, 48]

### 4.3 Isolation of EVs

The isolation and purification ofg EVs from biological fluids, necessary for clinical translation, present a major methodological challenge due to the natural variability in size, origin, and molecular composition of EVs, in addition to their coexistence in a complex mixture alongside contaminants like nucleic acid complexes, often share similar properties of size and density. Several isolation techniques have been developed, each impacting several parameters, including the amount of EVs recovered, their purity, and processing time. The conventional techniques comprise ultracentrifugation, size‐based separation, immunoaffinity capture, and polymer‐based precipitation. Recently, highly sophisticated fluidics‐based technologies were devised to boost enrichment efficiency and hasten the separation process [50, 51]. Ultracentrifugation is frequently used to isolate EVs. While it provides high yields and can be used to process large sample quantities, it requires long processing times of over 4 h [52, 53]. Size‐separation processes have been of interest as alternatives to centrifugation. Ultrafiltration uses membrane‐controlled pore sizes for high‐speed processing (under an hour), while size‐exclusion chromatography differentiates EVs based on size using porous media. Both these processes are faster and appropriate for bulk quantities, although they can lead to loss of vesicles due to adsorption on the membrane or pore face clogging problems [52]. Immunoaffinity capture methods offer the highest specificity by using antibodies that target certain EV surface markers or tumor‐specific markers. This technique is simple and necessitate a short time to isolate very pure EVs, though it is limited for processing small sample quantities [50, 52]. Polymer‐based precipitation methods, although simple, can introduce contaminants and disrupt EV structure and are associated with lower purity and yield, making them less ideal for applications that require the vesicles to function properly [52]. Microfluidic methods, a newer approach, use platforms that sort EVs from aggregates of proteins and cell waste mainly based on size differentiation. Although these technologies demonstrate better isolation efficiency compared to traditional approaches hampered by low throughput [50]. However, despite the developments in EV isolation techniques, existing methodologies are often affected by limitations such as low recovery rate and poor purity, which indicates the need for the development of more efficient methodologies with higher yield, greater homogeneity, reduced processing time, and decreased costs.

### 4.4 Characterization of EVs

For the characterization of EVs, accurate physical parameters like size, concentration, and morphology must be determined with certainty. Physical characteristics of EVs are mostly investigated using microscopic techniques. However, optical microscopes are incapable of obtaining high‐resolution images of EVs, since EVs are extremely small and optical microscopes are limited by their own diffraction limit. On the contrary, high‐resolution imaging techniques such as electron microscopy (EM) and atomic force microscopy (AFM) have been employed for collecting data underlying fine morphology. However, despite these techniques possessing increased resolution, they are throughput‐constrained by the requirement of special staining techniques and advanced equipment. Dynamic light scattering (DLS) represents another valuable technique for the study of several physical properties of EVs in suspension. It works based on measuring the scattered light by EVs after being excited using a monochromatic light. Because the vesicles are subject to Brownian motion, scattered light changes with time due to constructive and destructive interferences. The fluctuation rate is then used to determine particle diffusivity, allowing calculation of the hydrodynamic diameter using the Stokes–Einstein equation. However, DLS data must be carefully interpreted because it provides more weight to larger particles, which may distort the size distribution. Nanoparticle tracking analysis (NTA) offers an alternative to measure EV size and concentration. It tracks individual particles using light scattering, improving resolution for heterogeneous EV populations compared to DLS. However, NTA requires careful calibration of the camera and analysis settings, and sometimes multiple measurements are needed to capture all EV types in a sample. Tunable resistive pulse sensing (TRPS) is another technique that measures changes in ionic current as EVs pass via a size‐tunable nanopore. This allows for extracting data on the size and concentration of EVs. Together, these techniques provide useful information about EVs, although to get accurate and reliable results, it's important to consider the strengths and limitations of each method [50].

### 4.5 Stem‐Cell Based Nanocarriers

Stem cell‐based nanocarriers are a promising class of delivery system that incorporates stem cell therapeutics with nanotechnology accuracy. These systems mainly use two methods: nanoparticles coated with stem cell membranes and EVs derived from stem cells. Both approaches have unique benefits, providing more controllable substitutes to the conventional stem cell therapies [39].

#### 4.5.1 Stem Cell Membrane‐Nanocarriers

Cell membranes have long been used as a nanocarrier, resulting in the development of cell membrane‐coated nanoparticles as improved drug delivery tools. These bioinspired nanoparticles are characterized by better targeting ability and reduced immunogenicity. By integrating the complex functionalities of biological membranes and preserving the nanoscale dimensions and cargo‐loading capacity of nanocarriers, these systems provide a potential strategy for therapeutic applications. The fabrication process of these nanoparticles involves two principal steps, which are membrane vesicle isolation and subsequent fusion with core nanoparticles. Considering these concepts and advantages, stem cell membranes can be bioengineered to produce membrane‐encapsulated nanoparticles with improved targeted specificity and reduced immunogenicity. Among other sources, mesenchymal stem cell (MSC) membranes have emerged as a promising natural carrier through application of their encapsulating function and biocompatibility [38, 39].

#### 4.5.2 EVs as Drug Delivery Vehicles

EVs are increasingly used in neurodegenerative disorders, and stem cell‐derived EVs are at the forefront. They exhibit functions similar to their parent stem cells, with particular interest in neuronal regeneration, neuroprotection, immunomodulation, and the clearance of abnormal proteins. Recent research has also highlighted the growing therapeutic application of stem cell‐derived EVs due to their nanoscale dimensions and ability to encapsulate lipids, proteins, and nucleic acids, which allow for tissue repair in spinal cord injury (SCI). While unmodified EVs possess inherent therapeutic characteristics, engineered EVs have the benefit of increased efficacy by modulating their physicochemical properties, surface properties, and cargo. These developments improve delivery specificity to lesion locations, hence improving therapeutic effectiveness. Furthermore, EVs as drug nanocarriers provide a promising solution to the drawbacks of systemic drug administration, such as off‐target toxicities and side effects. As research on EV‐mediated drug delivery systems progresses, they are transitioning from the preclinical stage into clinical settings, offering a promising avenue for ND treatment. The biological origin of EV‐based nanocarriers, especially exosomes, allows them to evade immune detection and leverage natural transport pathways, giving them considerable superiority over synthetic nanoparticles when it comes to penetrating the BBB. In contrast to synthetic nanoparticles, which often rely on artificial surface modifications to overcome the immune system and cross the BBB, EVs possess natural surface proteins and lipids that allow for efficient, precise delivery to the brain. They also exhibit better biocompatibility, stability, cellular uptake and high specificity for target cells. Nevertheless, due to the complex pathophysiology of NDs, efficient EV‐based targeting remains challenging. As consequence, ample study has focused on developing EVs with specialized ligand–receptor interactions to improve cellular uptake and therapeutic efficacy in NDs. Various administration routes have been tested for EV‐based drug delivery, including intravenous, intracerebroventricular, intranasal, and oral. Intranasal administration has increasingly been identified as a noninvasive, efficient way of crossing the BBB via olfactory and trigeminal pathways, allowing for direct transport to the brain. Intranasal administration, however, can cause nonuniform EV distribution within the brain. Intracerebroventricular injections allow targeted delivery of EVs to a specific region of the brain but are invasive, making it clinically impractical. Every delivery strategy possesses specific advantages and limitations, which demand careful selection based on disease pathology, therapeutic objectives, patient safety, and treatment efficacy [52]. Consequently, the potential application of stem cell‐derived EVs—more specifically, exosome stem cells—for the treatment of NDs has been the centerpiece of this review.

## 5 Applications of Stem Cells in Neurological Disorders

**FIGURE 5:**
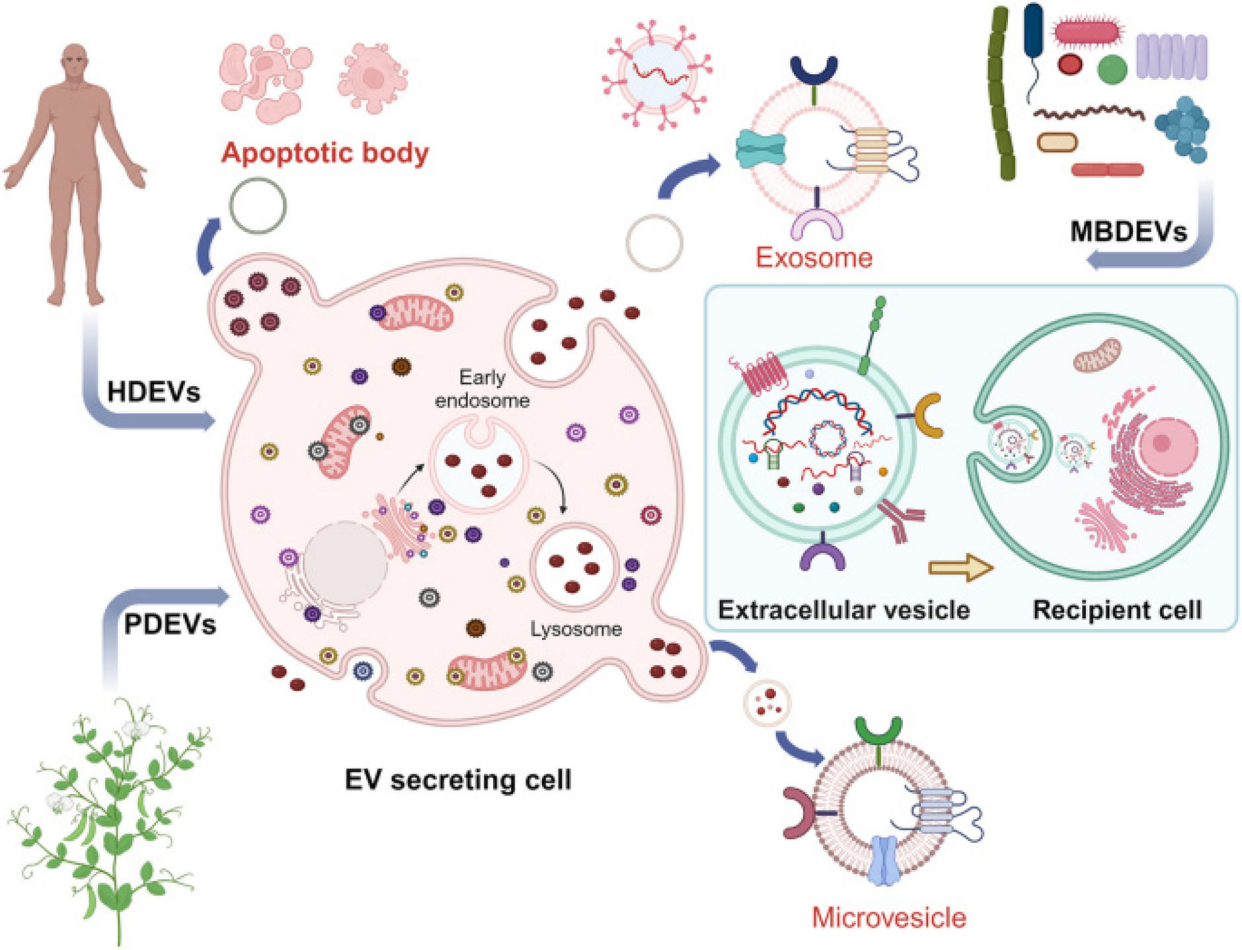
Graphical representation of diversity and sources of EVs. EVs are small‐scale particles secreted by humans (HDEVs), plant (PDEVs), and microbial (MBDEVs) cells, carrying bioactive molecules (proteins, nucleic acids, lipids) that mediate intercellular communication while maintaining source‐specific molecular signatures, highlighting their potential as therapeutic carriers and diagnostic tools across. Reproduce with permission from [54], Copyright @2025 Elsevier B.V.

Stem cell‐derived nanocarriers, especially exosomes (Figure 5), have shown significant potential as a novel therapeutic agent for a range of neurological conditions. Their distinctive features—including the capacity to penetrate the BBB, high biocompatibility, and suitability for targeted drug delivery—position them as effective candidates for managing complex neurological disorders. These EVs offer a noninvasive, cell‐free strategy that could contribute to mitigating critical pathological processes in AD. By decelerating or possibly preventing neurodegeneration and neuronal loss, EVs represent a novel and promising direction in AD therapy [55]. Beyond therapy, EVs also hold significant potential for AD diagnosis. Similar applications have been explored in other CNS disorders, including epilepsy, traumatic brain injury (TBI), and NDs, where EV‐based diagnostics show promise in identifying disease biomarkers and improving early detection.

### 5.1 AD

Exosomes offer a highly effective system for delivering therapeutic agents such as microRNAs, siRNAs, and antiamyloid compounds directly to neural tissues, aiding in the clearance of amyloid‐beta (Aβ) plaques, a key feature of AD. These vesicles, particularly those derived from stem cells, exhibit potent anti‐inflammatory and antioxidant effects that help counteract neurodegenerative processes. They can also transport neurotrophic factors, thereby enhancing neural connectivity and cognitive performance [56]. MSC‐derived EVs have received extensive attention in experimental models of neurodegeneration. For instance, Morris et al. developed an intranasal delivery of bone marrow MSC (BMSC)‐EVs to triple‐transgenic AD mice that decreased microglial activation and improved dendritic spine formation [57]. Similarly, studies in 5XFAD mice treated with EVs from three‐dimensional (3D)‐cultured BMSCs showed notable cognitive improvements along with decreased astrocyte reactivity and Aβ plaque accumulation [58].

In addition to bone marrow sources, EVs derived from adipose tissue MSCs rich in neuroprotective and regenerative proteins have proven beneficial in AD models. Intranasal administration of these EVs to APP/PS1 mice increased gene expression related to neurogenesis and neurite extension, while also insulating neurons from Aβ1‐42 oligomer and glutamate‐induced toxicity. Further research confirms the therapeutic efficacy of neural stem cell (NSC)‐derived EVs, which alleviated oxidative stress by decreasing intracellular reactive oxygen species (ROS) generation caused by 6‐hydroxydopamine (6‐OHDA), regulating inflammation, and preserving dopaminergic neurons [59]. Additionally, EVs sourced from human dental pulp stem cells significantly improved memory and spatial learning deficits caused by 6‐OHDA, with effects lasting several days. Improvements in motor function and gait were sustained even longer, persisting up to ten days post‐treatment [60]. Although stem cell‐derived EVs show considerable neuroprotective potential, the complexity of their cargo poses a challenge for consistency in therapeutic outcomes. A promising direction may be the modification of EVs to carry defined bioactive molecules, such as microRNAs, which can regulate numerous genetic pathways simultaneously. Their compact structure and robust activity make microRNAs (miRNAs) particularly promising for inclusion in next‐generation EV‐based therapies [61].

Although current evidence underscores the promising therapeutic value of EVs, additional research is necessary to uncover their precise biological mechanisms and refine their use in clinical settings. AD, specifically, continues to pose considerable problems in terms of early identification and treatment. Current diagnostic approaches rely heavily on identifying protein aggregation, which may not be visible in the early stages of the disease. In terms of treatment, just six FDA‐approved medications are available in the United States, none of which target the fundamental mechanisms driving the condition [62, 63]. Currently, there is no cure for AD, but only therapies that help manage symptoms [55]. As diagnosis and treatment remain complex and difficult to advance, the economic burden of AD continues to rise, with projections reaching approximately $1 trillion by 2050 [64]. This underscores an urgent need for innovative and effective scientific approaches to address both the medical and economic challenges posed by AD.

As scientific understanding advances, examining the surface proteins and internal contents of EVs obtained from patients may facilitate earlier and more precise diagnosis of AD by benchmarking these findings against validated reference data. Bioengineered EVs designed to target astrocytes and microglia may help mitigate AD pathology by reducing plaque accumulation, neuroinflammation, and vascular dysfunction [65, 66]. As cell‐free therapy, EV‐based treatments can circumvent the problems associated with stem cell therapies, such as cancer, uncontrolled cell proliferation, and immunological rejection. Planned clinical trials will be critical in determining the efficacy of nanoparticle‐based therapies for AD and other CNS conditions. More studies are essential to refine techniques for isolating EVs, improving their yield, enhancing tracking and targeting capabilities, and establishing reliable validation protocols to support their clinical translation.

### 5.2 PD

Dopaminergic Neuron Protection: Exosomes derived from MSCs can transport neuroprotective agents to protect dopaminergic neurons from oxidative stress and apoptosis. Alpha‐Synuclein Clearance: These nanocarriers may help degrade or prevent the aggregation of α‐synuclein, a toxic protein associated with PD. Exosomes carry anti‐inflammatory cytokines that can help reduce neuroinflammation in PD patients [67]. Because of the closely sealed endothelial cells and the restrictive properties of the BBB, the majority of large‐molecule drugs and more than 98% of small‐molecule drugs cannot penetrate it, severely restricting their therapeutic potential for neurological conditions [68]. Fortunately, emerging research has shown that EVs can cross the BBB via endocytic pathways, making them promising options for treating brain conditions. For instance, EVs encapsulating catalase (CAT) showed enhanced neuroprotective effects and oxidative stress reduction in PD models when compared to the enzyme in its free form, CAT [69]. Furthermore, intranasal delivery has proven effective in facilitating brain targeting of EVs, allowing passage either into systemic circulation through the nasal epithelium or directly into the brain via the olfactory and trigeminal pathways, effectively bypassing the BBB [70]. Zhuang et al. reported that fluorescently tagged EVs were broadly distributed throughout the brain as early as 30 min following intranasal delivery, indicating swift penetration and transport [71]. Notably, MSC‐derived EVs delivered intranasally displayed greater brain uptake at 1 and 24 h compared to intravenous treatment. Intranasal delivery offers several advantages, including rapid onset, noninvasiveness, and the ability to bypass hepatic and intestinal metabolism, ensuring high bioavailability. These benefits position it as a promising approach for treating CNS disorders, such as PD [69]. Intranasal EV administration has gained considerable interest in neurodegenerative disease research, particularly for PD and AD [69]. Peng et al. designed a self‐assembling nanocarrier system composed of a micellar core (PP@Cur) and a nanoshell (PR‐EXO), which integrates MSC‐derived exosomes with curcumin, collectively referred to as PR‐EXO/PP@Cur. A nanocarrier technology incorporating BMSC‐derived EVs and curcumin was found to increase membrane penetration and preferentially target injured dopaminergic neurons. This formulation, administered intranasally, improved motor skills and coordination in PD model mice by reducing α‐synuclein accumulation, enhancing neuronal performance, and decreasing neuroinflammatory reactions [72]. In a comparable vein, different research demonstrated that macrophage‐derived EVs accumulated higher in brain tissue within 4 h of intranasal treatment than intravenous delivery. Furthermore, giving catalase‐loaded EVs intranasally significantly decreased microglial activation in 6‐OHDA‐induced rats, providing substantial neuroprotection and an outcome not attained by free catalase [67].

### 5.3 TBI

Stem cell‐derived nanocarriers provide growth factors such as brain‐derived neurotrophic factors (BDNF) to assist neuronal repair and modulation of immunological responses by exosomes, collectively aiding the restoration of BBB integrity, which is normally compromised in TBI cases [73]. In an innovative technique, scientists created EVs by altering them with the CAQK peptide, a four‐amino‐acid sequence (Cysteine‐Alanine‐Glutamine‐Lysine) known for its propensity to selectively bind to wounded brain areas, notably in TBI. This targeting function was used for improved direct delivery of therapeutic drugs to the injured neurological regions. EVs were subsequently loaded with siRNA for the treatment of SCI, and the results showed that these modified vesicles greatly facilitated targeted repair and regeneration in traumatic SCI models [74]. Exosomes produced from adipose‐derived stem cells have been shown in recent in vivo studies to improve peripheral nerve regeneration after a crush injury. A recent study examined the effects of bone marrow‐derived MSC exosomes in a traumatic SCI model, demonstrating that exosome administration at various doses suppressed glial scar formation, decreased neuronal cell death and inflammatory responses, enhanced axonal regrowth, and significantly contributed to functional recovery following the acute phase [69]. Motor deficiencies of the limbs, ranging from weakness to complete paralysis, are among the most serious consequences of peripheral nerve injury (PNI), significantly impairing patients’ everyday lives and well‐being. Another study found that NSC‐derived exosomes strengthened neural tissue preservation and boosted functional recovery in a hemiplegia model [69], findings that were later corroborated by Liu et al. [75].

Investigations on TBI suggest that exosomes exert their beneficial effects primarily through paracrine signaling. MSC‐derived exosomes have been shown to enhance neurobehavioral recovery. Exosomes have been shown to promote neurogenesis and angiogenesis while lowering inflammation following TBI. A variety of cell types generate these therapeutic vesicles, including bone marrow‐derived stem cells (BMSCs), MSCs, olfactory ensheathing cells (OECs), NSCs, pericytes, and human umbilical cord MSCs (HUCMSCs). On the other hand, retro‐orbital infusion of BMSC‐derived exosomes in a controlled cortical impact (CCI) paradigm resulted in substantial increases in neurological function, as shown by modified neurologic severity scores (mNSS) at both 7 and 14 days post‐TBI [76]. Exosome‐treated animals showed better rotarod performance, decreased lesion volume, lower levels of apoptosis and inflammation, and a favorable shift in microglia/macrophage polarization from pro‐inflammatory M1 to neuroprotective M2. The M2 phenotype is known to assist axon regeneration and functional recovery in the CNS, adding to the therapeutic potential of exosomes in TBI [77].

Exosomes produced from MSCs have shown significant potential for treating various kinds of TBI. In a CCI model, these exosomes promoted spatial cognition as judged by the Morris Water Maze test and neurological efficiency as determined by the mNSS and forelimb foot fault tests. Furthermore, they increased vascular density, angiogenesis, and neurogenesis in the dentate gyrus while drastically lowering brain inflammation [78]. Exosomes from human MSCs have also been discovered to decrease neuroinflammation, leading to improvements in pattern separation function—a hippocampus‐dependent cognitive process—and spatial learning ability [79]. Exosomes’ role in miRNA secretion is a significant area of study. miRNAs are short noncoding RNAs that regulate gene expression post‐transcriptionally by binding to target mRNAs, causing destruction or translational suppression. Research demonstrates the importance of miRNAs in neuronal death, angiogenesis, immune‐inflammatory responses, and BBB permeability following TBI [78]. Pursuant to previously published investigations, miR‐21 levels peak 3 days after TBI and have been linked with enhanced neurological recovery, as demonstrated by improvements in mNSS and Morris Water Maze assessments. Likewise, higher miR‐124‐3p levels in microglial‐derived exosomes following repeated TBI (rTBI) have been identified to foster neurite extension and help recovery, possibly through the regulation of autophagy [80]. Several miRNAs, such as miR‐21‐5p, miR‐216a‐5p, and miR‐210, contribute to neuroprotection through increasing mitochondrial function and lowering lipid peroxidation in vascular endothelial cells [78]. Ultimately, stem cells’ therapeutic actions in TBI are due to secreted molecules, specifically exosomes. While preclinical results indicate excellent neuroprotective and functional recovery potential, more human research is needed.

### 5.4 Epilepsy

Exosome‐based therapies play a crucial role in mitigating oxidative stress and neuronal damage associated with recurrent seizures. By transporting specific microRNAs and proteins that regulate neuronal excitability, they can help modulate neuronal activity and potentially prevent seizure recurrence. In drug‐resistant epilepsy, exosomes offer a promising strategy by being engineered to transport antiepileptic drugs directly to the affected areas of the brain, providing a targeted method that could help overcome resistance to conventional therapies [81]. A significant barrier in employing iPSC‐derived neurons to model epilepsy is their limited microenvironment, which limits the creation of complex neuronal networks required to replicate seizure‐like activity. To address this issue, researchers grew iPSCs within 3D organoid systems that mimic early cortex development. These organoids can be generated through undirected protocols, allowing the formation of sophisticated neurological structures such as the retina, hindbrain, and cortical layers [82]. In epilepsy research, iPSC‐derived organoids have been applied to examine disorders such as Rett syndrome [83].

Animal models have traditionally played a critical role in advancing epilepsy research, but stem cell technologies, particularly iPSCs, provide a strong tool for modeling disease pathways and enabling high‐throughput screening (HTS) for patient‐specific treatment discoveries [84]. Current efforts in the induced pluripotent stem cell field center on improving differentiation methods to more precisely replicate disease pathology and accurately capture disease‐relevant phenotypes. Although iPSCs provide a robust basis for investigating genetic forms of epilepsy and have been increasingly used in autism spectrum disorder (ASD) research, further validation is needed to establish their reliability in predicting patient‐specific in vitro phenotypes. In addition to disease modeling, stem cell‐based therapies have shown potential in epilepsy treatment. Stem cell transplantation into mouse models has been found to be both safe and successful in reducing seizure frequency, highlighting their promise as a nondrug alternative for long‐term or chronic seizure control. Personalized therapeutics based on patient‐derived stem cells may transform epilepsy treatment and usher in a new era of precision medicine [84].

Implanting specific iPSC‐derived cell types into the epileptic brain allows for wide migration into numerous subfields of the target brain region, enhancing synaptic integration with the existing neural circuitry. Grafting mature GABAergic interneurons produced from human‐induced pluripotent stem cells (hiPSCs) into a mouse model of epilepsy has been demonstrated to reduce seizure activity while also enhancing cognitive function, mood, and aggressive behavior [85].

### 5.5 Other Neurological Disorders

Nerve fiber regeneration is crucial for restoring lost function following injury or disease. While the peripheral nervous system (PNS) retains an inherent capability to regenerate axons, recovery remains incomplete, particularly in cases of extensive or complex injuries. In contrast, nerve regeneration in the CNS is unsuccessful due to factors such as limited intrinsic regenerative capacity, neuronal apoptosis, scar formation, insufficient neurotrophic support, and inhibitory molecules derived from scars and myelin. CNS lesions, such as TBI, SCI, and optic nerve injury (ONI), present significant challenges in neuroregenerative research.

#### 5.5.1 PNI

The applications of stem cells have driven increasing research into their use for treating nervous system diseases, particularly given the limited capacity for neuronal regeneration. The therapeutic efficacy of stem cells depends on transplantation timing and the pathways involved, especially in PNI, where they have shown significant promise [86]. Studies in experimental stroke models have demonstrated that systemic stem cell administration can reduce brain injury, enhance neurological recovery, and also stimulate neurodegenerative mechanisms [87].

Current research suggests that stem cell‐derived EVs could serve as a more effective alternative to direct stem cell transplantation. These EVs provide various advantages, such as decreased immunogenicity, elimination of teratoma concerns, ease of administration, and the potential to target different parts of the nervous system other than the primary lesion site. MSCs have been demonstrated to modulate gene expression, inhibit autophagy, and exhibit antiapoptotic and anti‐inflammatory actions, particularly in restoring Schwann cell function [78]. Building on this research, a study investigated the effects of BMSC‐derived exosomes obtained under hypoxic conditions on Schwann cell proliferation in facial nerve injury, a type of PNI. Following on these findings, a study investigated the effect of BMSC‐derived exosomes collected in hypoxic circumstances on Schwann cell proliferation in facial nerve damage, an example of PNI. The results demonstrated that these exosomes improved Schwann cell proliferation and paracrine signaling via the circRNANkd2/miR‐214‐3p/MED19 pathway, indicating the possible involvement of stem cell‐derived EVs in nerve regeneration and functional recovery in NDs [88]. Further investigations emphasize the role of stem cell‐derived EVs in stimulating a favorable milieu for nerve regeneration, enhancing the release of neurotrophic and nerve growth factors, and therefore enabling neuronal repair and regeneration [89]. MSC‐derived EVs have therapeutic potential in PNI, including sciatic nerve damage, neuropathy, and partial or complete paralysis. These EVs modulate neuroinflammation, regulate the TSG‐6/NF‐κB/NLRP3 signaling pathway for immune responses, Schwann cell‐mediated processes via c‐Jun, and restore Neurotrophin‐3, an essential BDNF that supports neurogenesis [78]. With ongoing advancements, clinical studies on stem cell‐derived EVs continue to expand, positioning them as a promising, cost‐effective tool for neural regeneration [78]. While several in vivo and in vitro studies have established the therapeutic potential of MSC‐derived EVs in nerve injury, translating these findings into clinical applications remains difficult, and no definitive reports on their clinical success have yet been recorded.

#### 5.5.2 SCI

Currently, no treatment can fully regenerate axons following SCI. Significant strides have been made in understanding the barriers to CNS axon regeneration, leading to promising preclinical models [90]. Research in areas such as inflammation control, scar modulation, cell transplantation, and biomaterials has shown potential in promoting neural repair. More recently, combining stem cells, biomaterials, growth factors, and pharmacological agents has demonstrated greater success in axon regeneration compared to single treatments [91]. Although MSC‐derived EVs show strong therapeutic potential for SCI, several hurdles remain before they can be widely adopted. Under conventional culture conditions, stem cells tend to lose their regenerative properties and undergo senescence, reducing both their proliferation capacity and therapeutic efficacy [92]. Additionally, intravenously administered MSC‐EVs exhibit poor targeting to the injury site, which limits their effectiveness as SCI therapies [93]. Strategies to enhance MSC‐EV function include pretreatment under hypoxic conditions to preserve stem cell characteristics and boost proliferation. Furthermore, engineering modifications, such as piggybacking therapeutic agents onto EVs or modifying their membranes, may improve the targeting efficiency and therapeutic efficacy of MSC‐EVs for SCI treatment [78].

#### 5.5.3 ONI

ONI triggers fast degeneration of retinal ganglion cells (RGCs), which are responsible for the optic nerve's axons. Thus, RGCs have been the target of many neuroprotective therapy investigations, including antiapoptotic techniques such as anticaspase therapies [94, 95] and growth factors like BDNF [96]. However, EVs have lately emerged as a promising treatment option for eye injuries and disorders. According to research, MSC‐derived exosomes, particularly those carrying miRNA cargo, are essential for supporting RGC neuroprotection and axonal regeneration. Notably, inhibiting Argonaut 2 (Ago2), a critical regulator of miRNA activity, drastically reduces RGC neuroprotection and axon regeneration. ONI leads to fast degeneration of the optic nerve's RGCs [97]. Similarly, exosomes generated from bone marrow stem cells have been demonstrated to prevent retinal nerve fiber layer thinning and preserve function, but these benefits were significantly reduced when Ago2 was inhibited [98]. Furthermore, miRNAs modulate axon growth signaling pathways, which affect RGC regeneration and death. For example, miR‐93‐5p targets PTEN and modulates NMDA‐induced autophagy in RGCs during glaucoma [99]. Likewise, exosome‐mediated administration of the neuropeptide pituitary adenylate cyclase‐activating polypeptide 38 (PACAP38) was found to greatly improve RGC survival and axon regeneration [100]. Although most ONI research on exosomes focuses on their miRNA payload, the underlying mechanisms are still poorly known. Exosome composition varies depending on stem cell origin, contributing to variances in therapeutic benefits. To maximize EVs’ potential for treating ocular injuries and neurodegenerative eye disorders, a better understanding of their unique activities in ONI is required.

## 6 Current Advances and Preclinical Studies

Although in vitro and in vivo research on EVs is rapidly advancing, their therapeutic potential in people deserves careful consideration. Several completed and ongoing clinical trials are investigating EV uses for a variety of disorders. Therapeutic EV therapies may include unmodified EVs to analyze their natural effects or bioengineered EVs, which have demonstrated promising results in both preclinical and clinical investigations.

In recent years, only a limited number of clinical trials have focused on the therapeutic and diagnostic potential of EVs in NDs. One such study, NCT01860118, investigated EV biomarkers in the blood and urine of PD patients. While the results have not yet been disclosed, the research serves as a model for utilizing EV biomarkers to assess disease progression in other NDs. Monitoring EV proteome changes over time could yield important insights into the impact of aging and long‐term drug treatments on EV composition [55]. The current experiment, NCT04388982, evaluates the safety and efficacy of allogeneic adipose‐derived MSC EVs in Alzheimer's patients. NCT04388982 is a clinical trial assessing the safety and efficacy of allogeneic adipose‐derived MSC EVs in Alzheimer's patients. The study aims to assess cognitive performance, quality of life, and changes in Aβ levels in serum and CSF. Examining unmodified EVs in this situation may help us better understand their innate neuroprotective properties and ability to inhibit neuronal death. Similarly, NCT04040348 investigates the therapeutic potential of MSC infusions in patients with mild to moderate AD. This study will assess cognitive function, depressive symptoms, and overall quality of life for both patients and caregivers, as well as important blood biomarkers such as ApoE and tau protein. While MSCs are widely acknowledged to have considerable anti‐inflammatory and neuroprotective properties, concerns such as stem cell entrapment in the lungs and vasculature, as well as the risk of carcinogenesis, must be carefully addressed. In contrast, MSC‐derived EVs offer a promising, cell‐free alternative that bypasses these concerns while retaining therapeutic benefits. Another ongoing study, NCT04575337, is focused on collecting, detecting, and screening biomarkers across multiple stages of AD. By juxtaposing their findings, researchers may hope to create a biomarker panel for early AD diagnosis and risk prediction to those of age‐matched individuals with normal cognitive functioning [55]. These clinical studies highlight the growing interest in EVs as both diagnostic and treatment tools for NDs. Further investigations could pave the door for novel EV‐based therapies that improve early detection and treatment outcomes.

### 6.1 Challenges and Considerations in EV Therapy

To fully understand the impact of EV‐based therapies, it is essential to thoroughly evaluate long‐term regulation, the effects of repeated administration, and downstream biological consequences. While EVs are known to selectively target tissues in vivo, their sustained therapeutic effects remain under investigation. Notably, EVs demonstrate a longer half‐life compared to MSCs [101]. Recent advancements in NTA have enabled teams to study EV biodistribution. For instance, intravenously administered EVs tagged with fluorescently labeled CD63 in mice demonstrated rapid clearance, with a half‐life of under ten minutes [102]. However, robust analyses of EV half‐life in vivo remain limited [103]. Additionally, the mechanisms governing EV secretion and degradation remain poorly understood, and modifications to EVs could potentially affect their biodistribution and stability [55]. One of the key challenges in translating EV therapy to humans is the difficulty of generating sufficient EV quantities due to their heterogeneity. Standardized therapeutic applications require homogeneous EV populations, but isolation methods are time‐intensive, requiring extensive purification to obtain clinically relevant doses. This heterogeneity also affects the diagnostic potential of EVs, as their phenotypic profiles vary across samples, complicating their use as definitive biomarkers [104]. Recent advances like single‐EV sequencing and high‐sensitivity flow cytometry improve EV characterization, perhaps enabling more accurate selection based on desired features [105]. However, the field still faces an urgent need for standardized isolation methods and biomarker validation [104]. A major concern in NDs, particularly AD, is the prospect for EV‐based therapies to cause neurotoxicity or off‐target physiological reactions. Extensive tests are crucial and should be done in vitro and in vivo in animals before progressing to clinical application. Encouragingly, investigations have shown that autologous EVs do not induce neurotoxicity when readministered [55]. In PD research, EVs derived from murine blood, loaded with dopamine, and readministered to donor mice demonstrated reduced toxicity toward human neuroblastoma cells without inducing significant cell death in vivo [106]. While these findings are intriguing, further thorough testing will be required to ensure the safety and efficacy of new EV‐based therapies.

## 7 Limitations of Stem Cells in Neurological Disorder and Future Outlook

The broad application of cell‐based regenerative therapies is hampered by several practical challenges. A detailed awareness of these limitations and the incorporation of nanotechnology in addressing these issues will simplify the design and production of a combined product [107]. Traditional two‐dimensional (2D) static cultures, the first step in a variety of cell‐based therapies, require a labor‐intensive, time‐consuming, and clinical‐grade facility technique to differentiate a small number of cells. Additionally, evaluating the safety and efficacy of these cells as therapeutic agents is challenging due to their complexity. These difficulties include tumorigenicity, which addresses the potential for cell proliferation and the stability of its phenotype; mutagenesis, which may impact cell function; and contamination, which asks whether microbial sterility is guaranteed, as well as immunological activation or rejection [108, 109]. The requisite fundamental laboratory cell proliferation procedures would be challenging to verify, replicate, and guarantee quality control on a much more expansive scale, and it is expected that the manufacturing of sufficient numbers of these superior cells will not suffice for the demand [110]. Although these cells need artificial scaffolding to adhere to tissues once they’re in a system, the best model uses 3D cultures to form a functional tissue [111]. It is presently conceivable to create complex 3D dynamic cultures that replicate an in vivo environment using bioreactors that supply nutrients and oxygen, eradicate catabolites, monitor pH, and apply mechanical stress to stimulate the creation of an extracellular matrix [112]. Through injecting specialized growth agents, it is possible to maintain huge volumes of encapsulated undifferentiated cells in a bioreactor forever, directing their growth and differentiation and effectively replicating the physiological milieu unique to the tissue being regenerated [113]. Large‐scale bioreactors might mitigate batch sizes and unpredictability; however, additional study is required to investigate both viability and strategies for expanding production of cells [114]. Specialized cell types must also be generated for iPSC‐derived cell therapies since they require specific growth, transplantation, host integration, and genetic analysis setups to limit cancer risk. These elements present significant challenges to the effective enrichment or replacement of brain tissue; yet concurrent research is currently underway to identify the best approaches to stimulate or improve endogenous stem cells and their surroundings for optimal survival [115]. Another limiting aspect of stem cell transplantation therapy is immune rejection, which has a significant impact on their overall efficiency and efficacy [116].

Stem cell transplantation can induce immunological reactions that lead to the transplanted cells to be rejected, rendering the stem cell therapy ineffective [117, 118]. To lessen the chance of immunological rejection, experts are conducting extensive research into a range of approaches, including immune suppression medication and the delivery of patient‐specific stem cells. Despite a substantial corpus of research on the phenomenon of immune rejection, more study is required to increase its application in the context of stem cell therapy [117, 118, 119].

The ethical implications of stem cell therapy in clinical settings must be carefully considered. The following elements are mostly included in the ethical considerations. The origin of stem cells is a hotly debated ethical issue. Human embryonic stem cells are highly pluripotent and can develop into any type of cell in the body. However, because ESCs damage human embryos, there are ethical concerns about their utilization [120]. The other conceivable substitute is the study of iPSCs, which do not have the same ethical concerns [118, 121]. For every medical operation, including stem cell therapies, informed consent is a crucial ethical factor. After being fully informed of the risks and benefits of stem cell therapy, patients must provide their informed consent before commencing the course of therapy.

Access and Fairness: Issues of access and fairness may arise due to the cost and accessibility of stem cell treatments. Due to its high cost and potential inaccessibility to certain patients, stem cell therapies may result in unequal access to care. To sum up, comprehensive evaluation of ethical concerns is essential to guaranteeing that stem cell treatments are created and applied in a resp [118].

Safety, efficacy, and ethical issues are major regulatory obstacles for stem cell therapies deployed for brain regeneration [122]. The complex regulatory approval procedure calls for rigorous preclinical and clinical research while guaranteeing compliance with legal and ethical requirements. The development of safe and effective stem cell therapies for nerve regeneration is critically dependent on resolving these technological difficulties. Realizing that plenty more remains to be done, current research tasks are demonstrating considerable advances in addressing these barriers while propelling stem cell therapies toward realistic clinical applications [118]. Stem cell therapy and nanoparticles offer several opportunities to advance our knowledge regarding the oversight of NDs. One of the most fundamental challenges in treating neurodegeneration is a lack of understanding of the cellular pathways that drive lethal diseases. The lack of in vivo investigations is a major limitation in nanoparticle and stem cell research. Nanoparticles can be successfully employed to produce stem cells in vitro in highly regulated microenvironments, meeting the need for more accurate simulation systems for NDs. A substantial push for superior models will raise the cost‐effectiveness of possible drugs and reduce clinical trial failures [123].

More specifically, metal nanoparticles have enormous potential for both detecting and treating NDs. Presently, there are no current clinical trials treating NDs that combine stem cell treatment and nanoparticles. Combining metal nanoparticles and stem cell transplantation introduces exciting new possibilities for monitoring the disease's progression and therapy effectiveness [123]. The BBB poses a significant barrier, and the potential for unchecked development of transplanted stem cells renders its application in humans exceptionally risky. As a result, rather than injecting nanoparticles or stem cells into the brain, a more comprehensive therapy would be provided by first loading nanoparticles onto stem cells and then injecting them directly into the brain. Nanoparticles ought to optimally offer precise control over stem cell transplantation, the subsequent environment, stem cell development, and the repair of damaged surrounding cells [123]. Multiple techniques, including the creation of ROS, modification of signaling pathways, supervision of distinct components, and processes produced by metal nanoparticles, have been integrated for the synthesis and isolation of stem cells. Metal nanoparticles have a substantial effect on the concentration and isolation of stem cells; thus, it's critical to put into consideration any possible adverse effects both in vivo and in vitro [124]. Superparamagnetic iron oxide (SPIO) nanoparticles, such as SPIO‐Ferucarbotran, have been proposed to be an effective therapy for regenerative maladies. The superparamagnetic properties of SPIO nanoparticles enable them to be delivered to damaged regions and stimulate the growth of human MSCs by maintaining intracellular fluid and upregulating cell cycle‐related amino acids [124, 125, 126]. SPIO nanoparticles, like Fe_3_O_4_ and magnetic glazes, are distinguished by their strong magnetic dispersion moment, balanced biological properties, and low toxicity [125]. The enhancement and diversification of stem cells can be achieved by coating SPIOs with photonic zinc oxide and nanomaterial compounds, which promote noncovalent bindings of functional proteins and nanostructures. Modified core–shell nanoparticles with SPIO have been developed to regulate stem cell proliferation and transdifferentiation concurrently in vitro and in vivo [127]. iPSCs can be cultured on feeder layer cells to maintain pluripotency. Graphene and graphene oxide have recently been used as cell culturing substrates due to their relatively large 2D surface area and better biocompatibility at low concentrations. It was previously established that graphene/graphene oxide promotes discrete cell spreading and separation properties, as well as the capacity to harvest impulsive stem cell populations. The graphene oxide surface revealed a larger adherence and spread rate than glass surfaces, although iPSCs grown on graphene displayed comparable cell adhesion numbers and spread [124, 125]. Despite its potential for rapidly speeding up isolation, graphene has been found to be good at preserving iPSCs' uniformity. The development of cell–tissue interaction by integrating beneficial biological cellular signals with the physical microenvironment is critical for tissue engineering success. Consequently, more studies are needed to completely understand the kinetics of nanoparticle uptake and release in transplantable stem cells. Highly personalized therapy under continual supervision will substantially improve the success of ND remedies.

## 8 Conclusions

Stem cell nanocarriers are developing as a promising alternative neurotherapy due to their differentiation and self‐renewal performance to repair and regenerate damaged nerve cells. A wide range of stem cell types, including NSCs, induced neural stem cell iNSCs, PSCs, iPSCs, and MSCs, support neuron growth and have a beneficial effect on neural tissues by expressing neurotropic factors. Recent advances in cell‐based therapy, as well as the revelation of adult stem cell neurogenesis potential, have enabled novel approaches to neurologic treatment. NSC replication within the chipped neurons has contributed to their recovery. Implanting stem cells into a specific environment is one way to promote NSC growth. Implanted cell tumorigenicity, regulated stem cell differentiation, homogeneity, survivability, and continuing transplant cell tracking all pose significant challenges to this therapeutic strategy. These problems could be resolved with the advent of nanotechnology. A plethora of nanotechnology‐based approaches have emerged in recent years that have shown promise in medical research, especially as instruments for the identification and treatment of neurological conditions. Nanomaterials and stem cell cultures collaborate effectively to increase NSC replication, transmit functional compounds to target locations to promote NSC proliferation, and track the migration of implanted cells in real time, noninvasively, and over time. Despite this, every facet of utilizing nanoparticles requires to be assessed for possible negative effects and toxicity on a clinical level, comparable to any other technological advancement. Furthermore, the integration of stem cell‐mediated therapy, molecular biology, and nanotechnology may revolutionize these methods’ effectiveness and address their present problems. The effectiveness of conventional NSC‐based therapy for NDs is increased by molecular engineering of nanomaterials, subsequently rendering it practicable to deliver biological cargoes more precisely and track treated cells with greater accuracy.

## Author Contributions

**Nnamdi Ikemefuna Okafor**: conceptualization (lead), project administration (lead), visualization (equal), writing – original draft (equal), writing – review and editing (lead). **Ahmed Abdelgader**: conceptualization (supporting), visualization (equal), writing – review and editing (equal). **Magdi Abobaker**: writing – original draft (equal), writing – review and editing (equal). **Yahya E. Choonara**: supervision (lead), validation (lead), visualization (lead).

## Funding

This work was supported by the National Research Foundation (NRF) of South Africa (Grant No. PPNT230823145247).

## Conflicts of Interest

The authors declare no conflicts of interest.

## Data Availability

There is no data generated for this review.
